# Rates of glaucomatous visual field change before and after transscleral cyclophotocoagulation: a retrospective case series

**DOI:** 10.1186/s12886-015-0166-0

**Published:** 2015-12-14

**Authors:** Dominik Bleisch, Sandra Furrer, Jens Funk

**Affiliations:** Department of Ophthalmology, UniversityHospital of Zurich, Frauenklinikstrasse 24, Zurich, 8091 Switzerland

**Keywords:** Glaucoma, progression, rate of progression, visual field, transscleral cyclophotocoagulation

## Abstract

**Background:**

The primary goal of glaucoma treatment is to lower and control intraocular pressure (IOP) and thereby prevent functional deterioration. For glaucomas that are refractory to medical and incisional surgical therapies, transscleral diode cyclophotocoagulation (TCP) is a well-established procedure to effectively decrease IOP. This study investigated rates of visual field (VF) change in patients with glaucoma before and after TCP.

**Methods:**

This retrospective case series investigated rates of VF changes in glaucoma patients before and after they underwent TCP. At least four VF examinations were required, two before and two after surgery. VF examinations were performed using standard automated perimetry and rates of change were calculated by linear regression analysis of mean deviation (MD) values measured over time.

**Results:**

A total of 46 eyes of 43 patients were included and followed on average 3.6 years before and 2.1 years after TCP. 67 % of the eyes showed further progression of glaucoma following surgery. Mean preoperative MD change was −0.21 dB/year (SE = 0.08, 95 % CI [−0.06, −0.37]). Postoperatively the mean change was −0.26 dB/year (SE = 0.22 95 % CI [0.38, −0.48]) which results in a difference between pre- and postoperative MD rate of 0.05 dB/year (*p *= 0.824). The mean MD value was worse after surgery and dropped by 1.73 dB (SE = 0.58, 95 % CI [−0.59, −2.87], *p* = 0.003). Intraocular pressure (IOP) decreased from 23.2 mmHg (SD = 4.67) before TCP to 14.3 mmHg (SD = 3.17) after TCP (*p* < 0.001). For each 1 mmHg of IOP reduction after surgery, postoperative rate of VF loss decreased by 0.15 dB/year.

**Conclusion:**

Rates of glaucomatous visual field loss did not significantly change after TCP and the majority of the eyes showed further progression of glaucoma after surgery. Mean MD value was considerably lower after TCP.

## Background

Increased IOP has been identified as a major risk factor in the development and progression of glaucoma [1, 2]. Large prospective trials demonstrated the beneficial effect of reduced levels of IOP in delaying VF deterioration [3–5]. The primary goal of glaucoma treatment is therefore to effectively lower and control IOP and thereby prevent functional deterioration. This can either be achieved with medical therapy, incisional surgery or cyclodestructive methods such as TCP. For glaucomas that are refractory to medical and incisional surgical therapies, TCP is a well-established procedure and the effectiveness of the treatment on IOP reduction has been shown in various studies [6–10]. However, the final goal of glaucoma treatment remains the prevention of further VF deterioration as one of the most important factors for visual disability in patients with glaucoma. Unfortunately several investigations have shown that VF defects continue to progress in the majority of patients in routine clinical glaucoma care [11–14]. Obviously, the common treatment modalities are not able to completely halt VF progression, yet they play an important role in slowing it down. A number of studies have provided data on the changes in the VF after trabeculectomy [2, 15, 16]. However, data on VF changes after TCP has, to our knowledge, previously not been published. The purpose of the current study is to compare the rates of change in the VF before and after TCP in patients with difficult to manage glaucoma.

## Methods

### Subjects and data selection

This study retrospectively investigated the medical history of all glaucoma patients who underwent TCP at the University Hospital Zurich, Switzerland, between January 2007 and December 2012 (*n* = 388). The investigation was approved by the Regional Ethics Committee Zurich and conducted adhering to the tenets of the Declaration of Helsinki and in compliance with all local and national regulations and directives. Indications for TCP included uncontrolled glaucoma despite maximum tolerated medical treatment, drug allergy / intolerance, non-adherence to medication regimens or lack of response to filtration surgery. Patients were selected if two or more reliable VF examinations were available before and after TCP, either at our hospital or at the patients’ private ophthalmologist. All perimetric tests were performed with the Octopus G1/G2 program with dynamic strategy. VFs with test results showing more than 15 % false positive answers were excluded. Other exclusion criteria were filtration surgery or cataract extraction during follow-up, congenital glaucoma and other ophthalmic co-morbidity (except cataract) with serious impact on VFs. Patients with very low vision whose visual acuity was only classified with the semiquantitative scale "counting fingers", "hand motion", "light perception", and "no light perception" were excluded from the study. In order to analyse a trend in VF change the negative “Mean defect index” of every VF including the date of the examination was extracted from the Octopus printout. The MD change over time was calculated in decibel (dB) per year. Rates of VF changes were computed for the total sample of 46 eyes, for phakic and pseudophakic eyes separately and for each of the two glaucoma types “primary open-angle glaucoma (POAG)” and “pseudoexfoliation glaucoma (PEXG)” separately.

In order to quantify the effect of TCP on IOP we compared IOP measurements before and after surgery. Baseline IOP was defined as the mean of all IOP measurements performed within one month before the surgery. Postoperative IOP was determined as the mean of the last 3 measurements of the follow-up. If less than three IOP measurements were available, only two (11 eyes) or one (9 eyes) IOP value was taken into account. For seven eyes no postoperative IOP value was available and they were therefore excluded from the IOP analysis. Both baseline IOP and postoperative IOP were calculated from IOP values without any changes in the ocular hypotensive medications, and all measurements were performed using Goldmann applanation tonometry. According to the Guidelines on Design and Reporting of Glaucoma Surgical Trials [17] we defined treatment success as mean postoperative IOP between 4 and 20 mmHg, provided that medical hypotensive therapy was not increased.

As secondary endpoints, we analysed changes in medical hypotensive therapy and best-corrected visual acuity (BCVA). Before TCP, medical therapy was defined as the number of antiglaucoma medications used prior to surgery. Postoperatively, the number of medications was recorded at the last follow-up visit. If fixed combination medications were used, the number of active ingredients was documented. Altogether, medical therapy was obtained for a subgroup of 38 eyes. Preoperative BCVA was recorded from the last visit prior to surgery and postoperative BCVA was documented at the end of the follow-up. BCVA was analysed for a subgroup of 33 eyes.

Other data collected from the medical history and reported in this paper included gender, age, eye left or right, type of glaucoma, glaucoma surgery before follow-up, number and dates of all TCPs and lens status.

### Statistical methods

Data were recorded in Excel and analysed with SPSS Statistics Version 22 (IBM SPSS, Chicago, IL, USA). At patient level, descriptive statistics such as mean, standard deviation, minimum and maximum were computed for continuous variables, whereas absolute and relative frequencies were computed for discrete variables. Associations between age, gender and number of operations were analysed by Spearman rank correlation.

Association between MD and follow-up time corrected for pre- and postoperative, and interaction between postoperative follow-up time and pre-/postoperative status were computed. In order to account for imbalances in the number of VF per eye a linear mixed model was used [18]. The slope of the change in MD over time, together with a 95 % confidence interval for slope, were read up from the SPSS output. The MD change for each eye before and after TCP was calculated and displayed in a scatterplot. In addition, possible confounders such as gender and age were included in the analysis.

Mean values of IOP, medical hypotensive therapy and BCVA before and after TCP were compared using a paired t-test.

In order to investigate the influence of factors which are potentially associated with postoperative rates of VF changes, we performed a number of linear regression analysis with “MD at study start”, “preoperative MD change”, “IOP reduction”, “postoperative IOP” and “preoperative BCVA” as independent variables.

Results of statistical analysis with *p*-values smaller than 0.05 were considered to be statistically significant. Data more than 10 years before TCP was not eligible.

## Results

Out of 388 patient records, we obtained data from 43 patients (46 eyes) with a total of 421 VF examinations. Mean patient age at the time of TCP was 67.7 years with a range from 12 to 88 years. Mean preoperative follow-up time was 3.6 years, postoperatively patients were followed for 2.1 years on average. Table 1 shows detailed demographic data and other baseline characteristics.

**Table 1:** Demographics and baseline characteristics

	Mean (No)	SD	Median	Min	Max
Age (years)	67.7	14.1	68.8	12	88
Gender Female (%)	23 (50)				
Eye right (%)	27 (59)				
Treatment per eye	1.2	0.5	1	1	3
No. of preoperative VFs	5.5	3.3	5	2	14
No. of postoperative VFs	3.6	1.7	3	2	8
Last preop VF to surgery (days)	243.4	303.0	128	7	1421
Surgery to first postop VF (days)	273.2	138.6	269	16	596
Retreatment (%)	20				
Follow-up preoperative (years)	3.6	2.8	3	0.2	9.9
Follow-up postoperative (years)	2.1	1.4	1.7	0.4	5.8
IOP preoperative	23.2	4.7	23	14.4	33.2
IOP postoperative	14.3	3.2	14.1	8	22
No. of AGM preop.	3	1.5	3	0	6
No. of AGM postop.	2.44	1.42	3	0	6
BCVA preoperative	0.16	0.56	0.1	2.9	0
BCVA postoperative	0.22	0.52	0.15	2.9	−0.1
Phakic at the time of surgery (%)	23 (50)				
Surgical treatment before FU (%)	37 (80)				
– Trabeculectomy (%)	18 (39)				
– ELT (%)	4 (9)				
– SLT (%)	3 (6)				
– ALT (%)	4 (9)				
– Tube shunt (%)	2 (4)				
– Deep sclerectomy (%)	5 (11)				
– YAG–laser iridotomy (%)	1 (2)				

IOP, intraocular pressure in mmHg; AGM, antiglaucoma medications; BCVA, best–corrected visual acuity in logMAR; FU, follow–up; Preop, preoperative; Postop, postoperative; ELT, Excimer laser trabeculoplasty; SLT, Selective laser trabeculoplasty; ALT, Argon laser trabeculoplasty

**Fig. 1:**
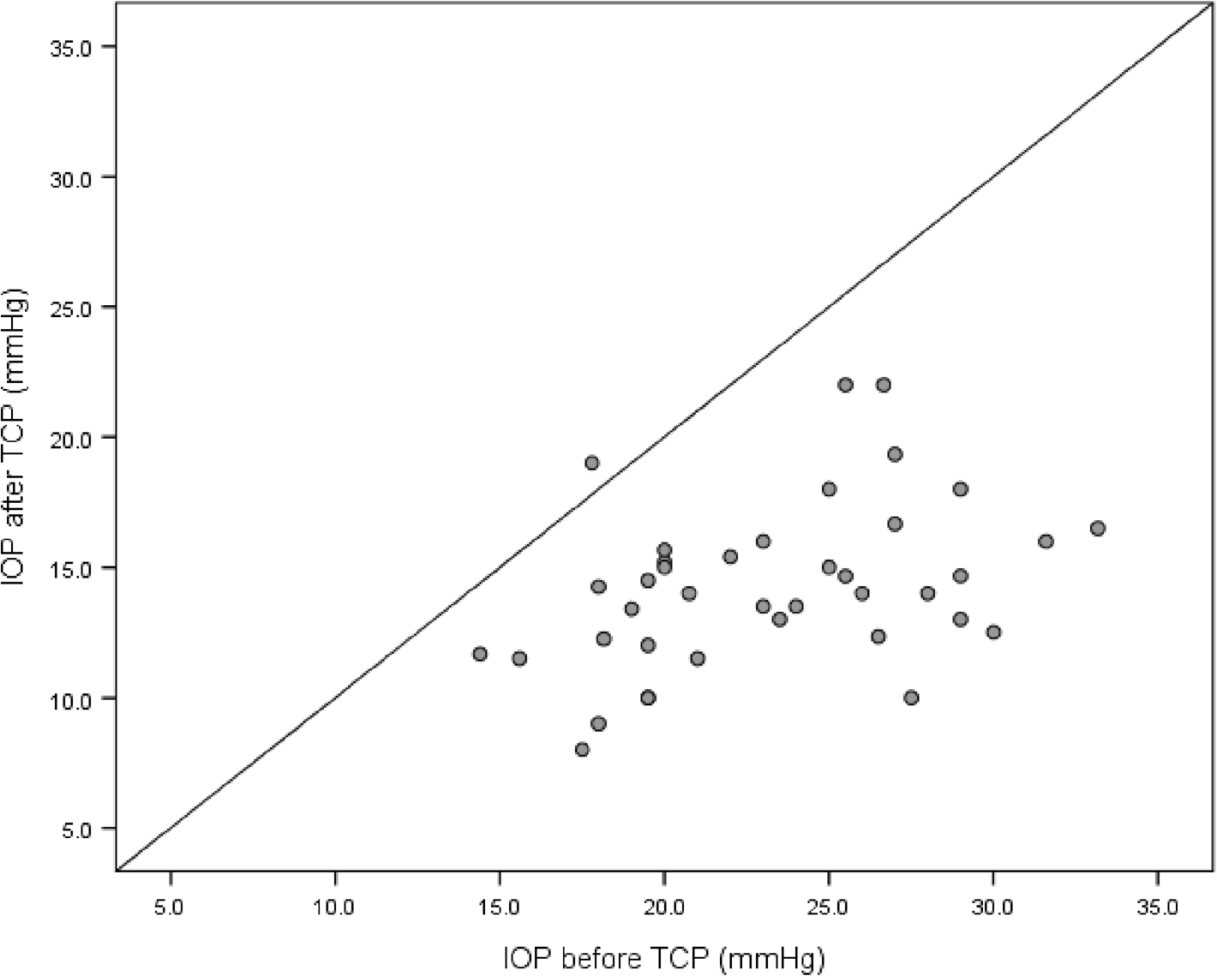
Average IOP in mmHg before and after TCP. Each dot represents an eye. Dots below the diagonal correspond to a decrease in IOP after surgery

After TCP, average IOP in the analysed subgroup of 39 eyes was reduced by 8.9 mmHg (*p*< 0.001, SD = 4.47), which corresponds to a relative reduction of 38 %. IOP reduction for each eye is illustrated in the scatterplot in Fig. 1. Success rate of the treatment, as defined earlier, was 82 % (31 of 38 eyes). Six eyes only achieved an IOP ≤ 20 mmHg with an increase of medical hypotensive therapy and in one eye IOP could not successfully be controlled.

Glaucoma types included in the study are displayed in Table 2. VF defects at the time of intervention covered the whole spectrum of disease severity with a mean MD of −9.6 dB (median 7.8; SD 6.8) and a range from −23.2 dB to +2.8 dB.

**Table 2:** Types of glaucoma included in the study

Glaucoma type	No	%
POAG	24	52
PEXG	12	26
Secondary glaucoma after trauma	2	5
Aphakic glaucoma	4	9
CACG	2	4.5
Other	2	4.5

POAG, primary open-angle glaucoma; PEXG, pseudoexfoliation glaucoma; CACG, chronic angle-closure glaucoma

**Fig. 2:**
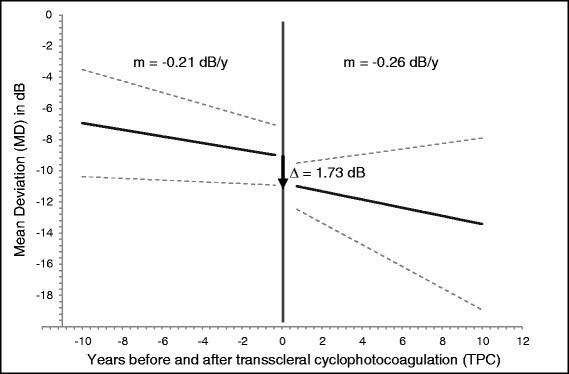
Average slopes (m) of VF loss in dB/year before and after TCP. The vertical double line indicates the moment of TCP (years = 0). The arrow marks the average MD loss (Δ) after TCP. Dashed lines represent the pointwise 95 % confidence interval for the average slope and the average MD loss after TCP

The mean rate of VF loss before TCP was −0.21 dB/year (SE = 0.08, 95 % CI [−0.06, −0.37]). After TCP the mean progression rate was −0.26 dB/year (SE = 0.22, 95 % CI [0.38, −0.48]) which results in a difference between pre- and postoperative MD change of 0.05 dB/year (*p* = 0.8235). The mean MD value dropped by 1.73 dB (SE = 0.58, 95 % CI [−0.59, −2.87], *p*= 0.00293) after TCP (Fig. 2). Adjusted for the variables age, gender and eye left/right, MD rate before TCP was −0.21 dB/year (SE = 0.10, 95 % CI [−0.01, −0.41]), after TCP −0.32 dB/year (SE = 0.20, 95 % CI [0.28, −0.49]) and the mean MD value dropped by 1.61 dB (SE = 0.52, 95 % CI [−0.59, −2.63], *p* = 0.00210). Rates of VF change for POAG, PEXG and phakic/pseudophakic eyes are listed in Table 3.

**Table 3:** Rates of visual field progression per type of glaucoma and lens status

	Before TCP (SE)	After TCP (SE)	∆	*p*
MDC POAG	−0.08 (0.07)	−0.09 (0.28)	0.01	0.966
MDC PEXG	−0.39 (0.06)	−0.81 (0.30)	0.42	0.164
MDC phakic	−0.10 (0.00)	−0.45 (0.26)	0.35	0.180
MDC pseudophakic	−0.28 (0.06)	0.03 (0.22)	0.31	0.168

MDC, mean deviation change in dB/year; SE, standard error; Δ, difference; POAG, primary open-angle glaucoma; PEXG, pseudoexfoliation glaucoma

Possible confounders were analysed and their individual effect on the VF loss was calculated. Neither age nor gender had a significant effect on the results.

The results of the linear regression analysis for the factors potentially associated with postoperative rates of VF changes are shown in Table 4. Larger IOP reduction was associated with significantly (*p*= 0.04) slower postoperative VF loss. For each 1 mmHg of IOP reduction after surgery, postoperative rate of VF loss decreased by 0.15 dB/year. For the variables MD change preoperative, BCVA preoperative and IOP postoperative, no significant correlation was found.

**Table 4:** Linear regression analysis of factors associated with postoperative rates of VF changes

	R^2^	β	Slope	*p*
MD at study start	0.0360	−0.1904	0.000	0.205
MD change preoperative	0.0007	0.0279	0.007	0.854
IOP reduction	0.1095	−0.3309	−0.150	0.040
IOP postoperative	0.0113	−0.1065	−0.120	0.519
BCVA preoperative	0.0010	−0.0330	−0.000	0.844

MD, mean deviation in dB; IOP, intraocular pressure in mmHg; BCVA, best-corrected visual acuity in logMAR

**Fig. 3:**
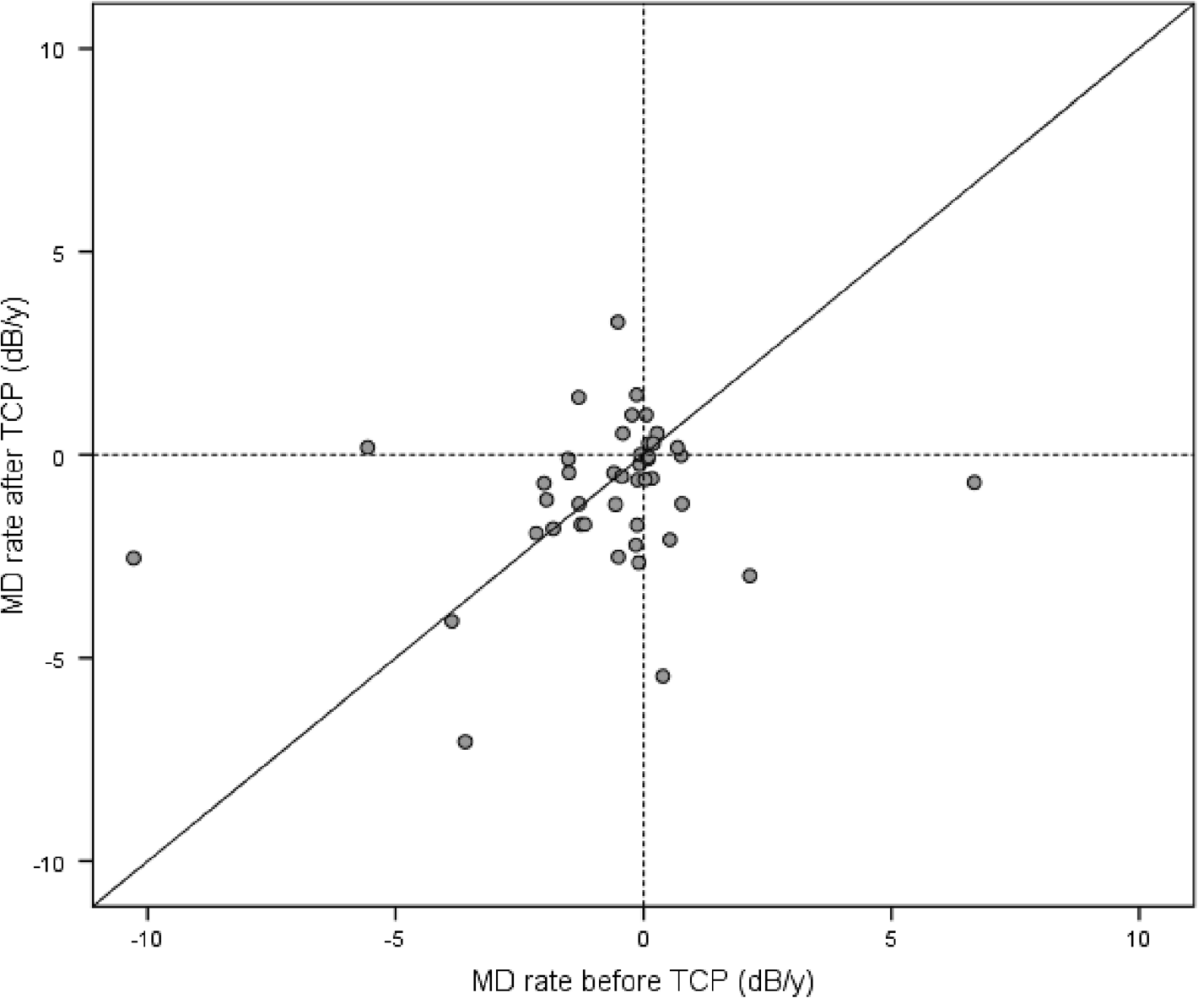
MD rate in dB/year for each eye before and after TCP. Dots above the diagonal correspond to a slowing of VF loss after TCP whereas dots below refer to an accelerated VF loss after surgery

Progression rates on the eye level are illustrated with a scatterplot in Fig. 3. 31 of 46 eyes (67 %) showed further progression of glaucoma after surgery. Among them, nine eyes showed a deceleration in VF loss whereas 22 eyes progressed on a faster rate following laser treatment. Only a minority of 15 eyes improved after TCP. The analysis with a Spearman rank correlation showed no significant (*ρ* = 0.148, *p* = 0.326) association between pre- and postoperative MD rate.

## Discussion

The main goal of glaucoma treatment is to effectively lower and control IOP and prevent further deterioration of the VF. Data from the Advanced Glaucoma Intervention Study showed that low IOP is associated with reduced progression of VF defects, supporting evidence from earlier studies of a protective role of low IOP in VF deterioration [4]. The 38 % relative IOP reduction found in this study is in accordance with numerous other studies demonstrating the effectiveness of TCP in significantly reducing IOP [6–10]. Our investigation on the rates of glaucomatous VF change before and after TCP showed, during a mean preoperative follow-up time of 3.6 years, an MD rate of −0.21 dB/year (SE = 0.08, 95 % CI [−0.06, −0.37]). This VF loss is in agreement with a study from Chauhan et al. [12] that reported a median MD rate of −0.05 (interquartile range 0.13, −0.3) in patients under routine glaucoma care. After TCP the rate of VF loss increased by 0.05 dB/year (*p* = 0.8235) to an MD rate of −0.26 dB/year (SE = 0.22 95 % CI [0.38, −0.48]) over a mean postoperative follow-up time of 2.1 years. We found that larger IOP reduction was associated with a significant (*p* = 0.04) decrease in postoperative rate of VF loss. For each 1 mmHg of IOP reduction after surgery, postoperative rate of VF loss decreased by 0.15 dB/year. Despite good IOP lowering and a significant association between IOP reduction and postoperative progression rates, our study showed that TCP could not halt glaucomatous VF progression in our group of patients and in the majority of the eyes (67 %), VF loss continued after surgery. Even though it was not significant, this change is in contradiction with previous studies which reported improved MD rates after glaucoma filtration surgery with a comparable relative IOP reduction [15, 16]. Folgar et al. [16] found that mean global progression rates significantly decreased from −1.48 dB/year (SD 1.4) before surgery to −0.43 dB/year (SD 0.8) after surgery. Each 1 mmHg of IOP reduction after trabeculectomy resulted in a 0.1 dB/year decrease in the global rate of progression. Bertrand et al. [15] found an average difference between the rates of MD loss of 0.20 dB/year (*p* = 0.15).

The significant drop in mean MD value after TCP is striking. The loss of visual acuity after TCP is a common complication and could explain deteriorations in the VF [7, 10]. Since we only documented a post-operative BCVA loss of 0.06 logMAR, loss of visual acuity can hardly be responsible for the damage in the VF. The lower number of VF examinations after TCP (3.6 after vs. 5.5 before TCP) could be another source of error. Besides the quality of each examination, the validity of MD values strongly depends on the number of VF tests performed. Chauhan et al. [19] showed that a total MD change of −2 dB over a 3 year period requires 4 annual examinations in order to detect the change with 80 % power. Although VF examinations where performed by many different private ophthalmologists, testing conditions before and after TCP were identical for each patient since they remained at the same private practice during the entire time they were followed. Examination quality was ensured by excluding VF examinations with more than 15 % false positive answers.

Cataract might have played a role in the postoperative worsening of the VF since we found that phakic eyes progressed considerably faster after TCP than pseudophakic eyes (−0.45 dB/y versus 0.03 dB/y). Even though this difference was not statistically significant, it indicates that lens opacification probably contributed to the lack of reduction in VF loss after surgery.

Our relatively large proportion of PEXG could be another reason for the VF loss after TCP. We found that MD loss after surgery increased substantially among patients with PEXG compared to almost no increase among POAG patients (0.42 dB/y versus 0.01 dB/y). Earlier studies suggested that pseudoexfoliation is a strong factor for progression of glaucoma and it is possible that natural progression of the disease is responsible for the increased MD loss [3, 20].

The main limitation of our study is its retrospective nature. Since our hospital is a referral centre and many patients return to their private ophthalmologists after surgical treatment, data has not only been collected at our hospital but was obtained from several referring ophthalmologists. Our findings should therefore be interpreted with caution.

## Conclusion

Our study shows that rates of glaucomatous VF loss did not significantly change after TCP. Despite a successful IOP lowering, the majority of the eyes showed further progression of glaucoma after surgery. To our knowledge, this study is the first published investigation on the rate of VF change before and after TCP and adds further insights in the progression of VF defects after invasive IOP lowering. It remains unclear why rates of glaucomatous VF progression after TCP were not reduced and whether the postoperative lower baseline MD value results from treatment or from the natural progression of glaucoma.

## Abbreviations

IOP: Intraocular pressure
TCP: Transscleral diode cyclophotocoagulation
VF: Visual field
MD: Mean deviation
MDC: Mean deviation change
POAG: Primary open-angle glaucoma
PEXG: Pseudoexfoliation glaucoma
CACG: Chronic angle-closure glaucoma
BCVA: Best-corrected visual acuity
AGM: Antiglaucoma medications
FU: Follow-up
Preop: Preoperative
Postop: Postoperative
ELT: Excimer laser trabeculoplasty
SLT: Selective laser trabeculoplasty
ALT: Argon laser trabeculoplasty
